# Characterizing the clinical heterogeneity of early symptomatic Alzheimer’s disease: a data-driven machine learning approach

**DOI:** 10.3389/fnagi.2024.1410544

**Published:** 2024-08-12

**Authors:** Xiwu Wang, Teng Ye, Deguo Jiang, Wenjun Zhou, Jie Zhang, for the Alzheimer’s Disease Neuroimaging Initiative

**Affiliations:** ^1^Department of Psychiatry, Wenzhou Seventh People’s Hospital, Wenzhou, China; ^2^Department of Ultrasound, The First Affiliated Hospital of Wenzhou Medical University, Wenzhou, China; ^3^Research and Development, Hangzhou Shansier Medical Technologies Co., Ltd., Hangzhou, China; ^4^Department of Data Science, Hangzhou Shansier Medical Technologies Co., Ltd., Hangzhou, China

**Keywords:** Alzheimer’s disease, heterogeneity, cognitive trajectories, longitudinal clustering, subtypes

## Abstract

**Introduction:**

Alzheimer’s disease (AD) is highly heterogeneous, with substantial individual variabilities in clinical progression and neurobiology. Amyloid deposition has been thought to drive cognitive decline and thus a major contributor to the variations in cognitive deterioration in AD. However, the clinical heterogeneity of patients with early symptomatic AD (mild cognitive impairment or mild dementia due to AD) already with evidence of amyloid abnormality in the brain is still unknown.

**Methods:**

Participants with a baseline diagnosis of mild cognitive impairment or mild dementia, a positive amyloid-PET scan, and more than one follow-up Alzheimer’s Disease Assessment Scale-Cognitive Subscale-13 (ADAS-Cog-13) administration within a period of 5-year follow-up were selected from the Alzheimer’s Disease Neuroimaging Initiative database (*n* = 421; age = 73±7; years of education = 16 ± 3; percentage of female gender = 43%; distribution of APOE4 carriers = 68%). A non-parametric k-means longitudinal clustering analysis in the context of the ADAS-Cog-13 data was performed to identify cognitive subtypes.

**Results:**

We found a highly variable profile of cognitive decline among patients with early AD and identified 4 clusters characterized by distinct rates of cognitive progression. Among the groups there were significant differences in the magnitude of rates of changes in other cognitive and functional outcomes, clinical progression from mild cognitive impairment to dementia, and changes in markers presumed to reflect neurodegeneration and neuronal injury. A nomogram based on a simplified logistic regression model predicted steep cognitive trajectory with an AUC of 0.912 (95% CI: 0.88 – 0.94). Simulation of clinical trials suggested that the incorporation of the nomogram into enrichment strategies would reduce the required sample sizes from 926.8 (95% CI: 822.6 – 1057.5) to 400.9 (95% CI: 306.9 – 516.8).

**Discussion:**

Our findings show usefulness in the stratification of patients in early AD and may thus increase the chances of finding a treatment for future AD clinical trials.

## Introduction

Alzheimer’s disease (AD) is a heterogeneous disorder with high individual variabilities in cognitive progression (Jutten et al., 2021; Cohen et al., 2024). In the search for treatment, people have so far predominantly targeted one single clinical entity and assumed that individuals are homogeneous in the course of cognitive decline and the placebo and treatment groups should demonstrate equal rates of cognitive deterioration if the treatment is ineffective (Fogel, 2018). Nevertheless, several longitudinal studies have found substantial variations in the rates of cognitive decline among patients with AD despite being matched for clinical severity at the beginning of the study (Jack et al., 2010b; van Rossum et al., 2012; Vos et al., 2015; Scheltens et al., 2018). A consequence is that cognitive heterogeneity may bias the results of therapeutic clinical trials of AD. For instance, even though within most previous trials no difference between placebo and treatment groups was observed, we cannot fully rule out the probability that the null effect was actually attributed to over-representation of individuals with slow rates of cognitive decline in the treatment group or over-representation of individuals with rapid rates of cognitive decline in the placebo group, if treatments were actually effective (Jutten et al., 2021). Moreover, a specific therapeutic strategy may only benefit certain subgroups of patients, and thus identification of meaningful subgroups of individuals with AD may be a crucial first step towards improving the clinical trial design, increasing the chance of finding an efficacious treatment and developing personalized medicine.

One strategy to identify subtypes is to categorize patients based on cognitive features using empirical methods in a non-biased manner (Gamberger et al., 2017; Lee et al., 2018; Kim et al., 2019, 2022; Blanken et al., 2020; Edmonds et al., 2021; Giraldo et al., 2021; Wang et al., 2023; Kim B. S. et al., 2023; Kim Y. J. et al., 2023). For cross-sectional neuropsychological data, several previous studies utilized a data-driven approach to sort out the cognitive heterogeneity of AD by applying clustering methods, leading to the identification of differential cognitive subtypes (Scheltens et al., 2015, 2017; Qiu et al., 2019). However, cross-sectional cognitive data can only capture the heterogeneity in cognitive deficits at a snapshot and are unable to delineate the temporal nature of disease progression. For these reasons, several investigators (Geifman et al., 2017; Ziegler et al., 2020; Levine et al., 2021) have undertaken subtyping approaches to understand the heterogeneity of AD by using repeatedly measured cognitive outcomes, namely Alzheimer’s Disease Assessment Scale-Cognitive Subscale (ADAS-Cog), which is a commonly used primary endpoint in clinical trials of AD. In Geifman et al.’s study (Geifman et al., 2017), latent class mixed modeling (LCMM) has been conducted to successfully identify 3 distinct longitudinal cognitive subgroups (i.e., rapid decliners, slow decliners, and severely-impaired slow decliners) over a period of 18 months in a clinical trial database involving clinically diagnosed AD dementia patients (Geifman et al., 2017). In Ziegler et al.’s study (Ziegler et al., 2020), a statistical clustering method has been performed to identify 3 different cognitive subgroups (i.e., “mild impairment” group, “memory impaired” group, and “fast progressing” group) over a period of 2 years among patients with a clinical diagnosis of AD dementia. More recently, by leveraging data from five clinical trials of donepezil for AD patients, Levine et al. (2021) performed a latent class model to identify 3 subgroups (i.e., low scorers, improvers, and high scorers) over a period of 12 weeks. Nevertheless, despite the shift from a syndromal definition to a biological definition of AD in observational and interventional research, and AD clinical trials are moving earlier in the disease process (Jack et al., 2018), no studies have yet investigated the cognitive heterogeneity of early symptomatic AD [mild cognitive impairment (MCI) or mild dementia due to AD] with evidence of abnormal amyloid in the brain. We undertook such a study, using repeatedly measured longitudinal cognitive outcomes over a period of 5 years to identify classifications of patients with early AD that show distinct clinical progression trajectories.

In this study, we expanded upon previous research by applying a state-of-the-art statistical clustering method to longitudinal ADAS-Cog-13 data collected within a 5-year period from patients with early AD who showed evidence of abnormal amyloid and met inclusion and exclusion criteria commonly applied in a typical clinical trial involving patients with early AD (van Dyck et al., 2023). Following the clustering analysis, we examined associations of the identified cognitive trajectories with changes in other cognitive and functional measures, neurogenerative biomarkers, *in vivo* CSF AD biomarkers, and disease progression to AD dementia. We investigated potential baseline predictors that may be associated with membership in the identified subtypes. Finally, a nomogram was created to facilitate an easy and practical prediction of probabilities of experiencing steep cognitive decline, and simulated clinical trials were conducted to examine whether the incorporation of the nomogram into the enrichment strategy would lead to the reduction of sample size for trials involving early AD.

## Materials and methods

### Study participants

Data were obtained from the Alzheimer’s Disease Neuroimaging Initiative (ADNI) database.^1^ ADNI is a multicenter longitudinal cohort study with the main research goal of examining whether clinical, neuropsychological, biological, and other neuroimaging markers can be combined to track clinical progression in the Alzheimer’s disease continuum. The ADNI study was approved by an ethical review board of participating study centers and all subjects provided written informed consent.

**Figure 1:**
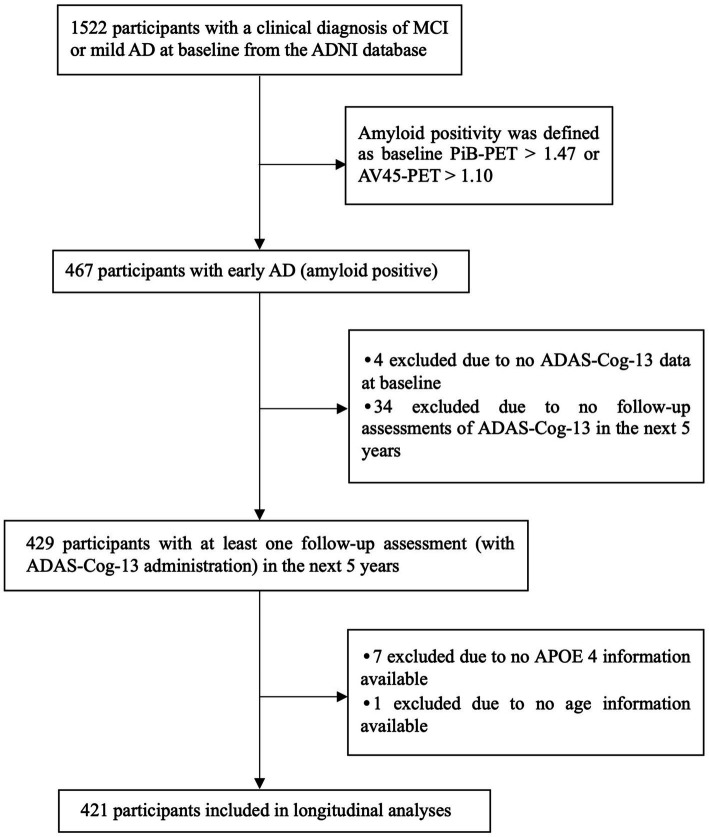
Flowchart showing the sample selection procedure. MCI, Mild cognitive impairment; AD, Alzheimer’s disease; PiB, Pittsburgh compound B; AV45, Florbetapir; ADAS-Cog-13, Alzheimer’s Disease Assessment Scale-Cognitive Subscale 13; APOE, Apolipoprotein E.

For the current study, we selected subjects with a baseline clinical diagnosis of either MCI or mild AD dementia who had elevated amyloid as determined by PET imaging (specific methods and cutoff described below) and had at least 1 follow-up measurement available (with ADAS-Cog-13 administration) the next 5 years. Criteria for MCI were (1) memory complaint; (2) abnormal memory function evidenced by the Logical Memory II subscale (Delayed Paragraph Recall) from the Wechsler Memory Scale-Revised; (3) Mini-Mental State Examination (MMSE) score ≥ 24; (4) global Clinical Dementia Rating (CDR) score of 0.5; (5) absence of dementia. Criteria for mild AD dementia were (1) memory complaint; (2) abnormal memory function evidenced by the Logical Memory II subscale from the Wechsler Memory Scale-Revised; (3) MMSE score between 20–26 (inclusive); (4) global CDR score of 0.5 or 1; (5) NINCDS/ADRDA criteria for probable AD dementia (Mckhann et al., 1984). This study included 421 participants with early AD (MCI or mild dementia due to AD). The inclusion criteria for participants with early AD in the current study largely aligned with those used in a recent anti-amyloid AD clinical trial (van Dyck et al., 2023). For detailed sample selection procedures, please see Figure 1.

### Clustering cognitive variable

The ADAS-Cog (Rosen et al., 1984) is a commonly used cognitive outcome for tracking disease progression and measuring the efficacy of antidementia treatments and the most used cognitive outcome measure in AD clinical trials (Cano et al., 2010). Thus, we treated ADAS-Cog as our primary cognitive outcome and used it as the variable for longitudinal cluster analysis (specific procedures for cluster analysis described below). The 13-item version of ADAS-Cog (ADAS-Cog-13) includes 13 tasks that primarily assess the cognitive domains of episodic memory, praxis, and language. Total score ranges from 0 to 85, with higher scores indicating greater cognitive impairment (Rosen et al., 1984).

### Other cognitive measures

Apart from the ADAS-Cog, the second and third most frequently used cognitive outcome measures in AD clinical trials were the MMSE (Folstein et al., 1975) and the CDR-sum of boxes (CDR-SB) (Williams et al., 2013; Jutten et al., 2021). The Functional Activities Questionnaire (FAQ) (Pfeffer et al., 1982) is a commonly used instrumental activities of daily living (IADLs) scale that predicts clinical progression (Marshall et al., 2015). Therefore, these cognitive measures (i.e., MMSE, CDR-SB, and FAQ) were taken to assess and validate cognitive and functional changes over time between different cognitive subgroups. The MMSE is a widely used global cognitive screening test, with total scores ranging from 0 to 30, and a lower score indicates greater cognitive impairment (Folstein et al., 1975). The CDR-SB captures 6 cognitive and functional domains, including memory, orientation, judgment and problem-solving, community affairs, home and hobbies, and personal care (Williams et al., 2013). Scores for each domain range from 0 to 3, with higher scores reflecting greater impairment. Adding scores of each domain leads to a total score, which ranges from 0 to 18 (Williams et al., 2013). The FAQ rates 10 functional domains, with total scores ranging from 0 to 30, and higher scores indicate greater impairment (Pfeffer et al., 1982).

### Determination of amyloid positivity

Amyloid status (amyloid negative vs. amyloid positive) was determined based on Pittsburgh compound B (PiB) or Florbetapir AV-45 PET imaging (summarized data were pulled from the ADNI Laboratory of Neuroimaging database: ida.loni.usc.edu; the file name is ADNIMERGE.csv). Amyloid positivity was determined by calculating the standardized uptake value ratio of the mean uptake in four cortical regions (frontal, cingulate, parietal, and temporal cortices), which was normalized to the uptake in the entire cerebellum. We used validated tracer-specific cutoff values to determine abnormality, which were > 1.47 for PiB-PET and > 1.10 for AV45-PET (Landau et al., 2013).

### Neurodegenerative markers

Hippocampal atrophy, ventricular enlargement, and cerebral glucose hypometabolism are presumed to reflect neurodegenerative alterations most proximal to the onset of cognitive decline, and valid predictors of disease progression (Jack et al., 2004, 2010a). Therefore, we took these three imaging markers to characterize neurodegenerative changes among cognitive subgroups. Summary data were obtained from the ADNI Laboratory of Neuroimaging database: ida.loni.usc.edu; the file name is ADNIMERGE.csv. The proportional approach [(normalization of regional volumes by intracranial volume (ICV)] was taken to adjust sex differences in head size since women and men differ substantially in ICV. Adjusted ventricular volume (aVV) was calculated using the following equation: aVV = ventricular/intracranial volume × 10^3^. Adjusted hippocampal volume (aHV) was calculated using the following equation: aHV = hippocampal/intracranial volume × 10^3^. Cerebral glucose metabolism was measured by Fludeoxyglucose PET (FDG-PET). The global FDG SUVRs were measured by calculating the mean FDG uptake in three brain regions (posterior cingulate, angular gyri, and inferior temporal gyri), which was normalized to the uptake in the pons and cerebellum.

### CSF AD biological markers

Lumbar puncture was conducted as described in the ADNI manual.^2^ The levels of CSF AD biomarkers, including CSF Aβ 1–42 (Aβ42), total tau (t-tau), and phosphorylated-tau at threonine 181 (p-tau), were measured by the Roche Elecsys Aβ42 CSF, Elecsys t-tau CSF, and Elecsys p-tau CSF immunoassays at the Biomarker Research Laboratory, University of Pennsylvania, USA, as previously described (Bittner et al., 2016). In this study, Elecsys Aβ42 values >1700 pg./mL (upper technical limit) were fixed at 1700 pg./mL. CSF Aβ42 values below 1,098 pg./mL were used to classify individuals as amyloid-positive, based on thresholds established in a previous study (Schindler et al., 2018).

### Statistical analyses

Statistical work and data visualization were conducted using R version 4.1.2 (Team, R.Core, 2014).

**Figure 2:**
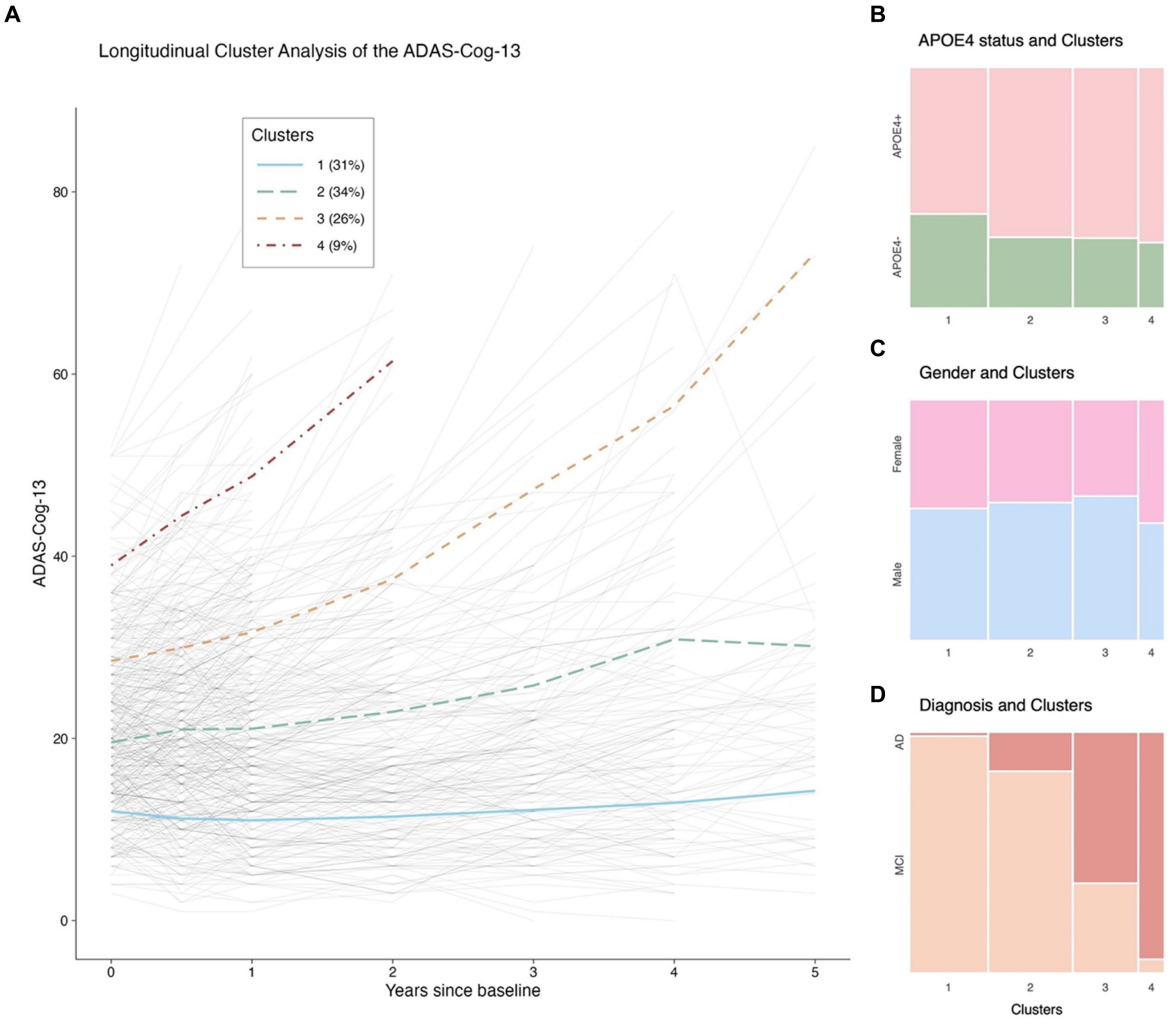
.The ADAS-Cog-13 trajectories from baseline to 5 years and their relations with APOE 4 genotype, gender, and clinical diagnosis. Panel **(A)**: Longitudinal cluster analysis of the ADAS-Cog-13 scores. Panel **(B)**: Relationship between APOE4 status and Clusters. Panel **(C)**: Relationship between gender and Clusters. Panel **(D)**: Relationship between diagnostic status and Clusters. Notes: The solid blue, long-dash green, dash orange, and dot-dash red lines in **(A)** represent Clusters 1, 2, 3, and 4, respectively. The thin gray lines represent individual trajectories. Abbreviations: ADAS-Cog-13: Alzheimer’s Disease Assessment Scale-Cognitive Subscale 13; APOE: Apolipoprotein E; MCI: Mild cognitive impairment; AD: Alzheimer’s disease.

First, to identify distinct longitudinal cognitive profiles, a non-parametric k-means longitudinal clustering method from the R package “kml” (Genolini et al., 2015) was used to detect the ADAS-Cog-13 trajectories over a 5-year follow-up period. This method allows for the investigation of how a parameter of interest changes over time and categorizes individual trajectories into distinct groups of participants with homogeneous characteristics. The ADAS-Cog-13 was treated as our clustering variable given its predominant role in AD clinical trials. In the cluster analysis, we only included participants who had undergone at least one follow-up assessment using ADAS-Cog-13 within 5 years since baseline. K-means is an algorithm in the expectation–maximization (EM) (Celeux and Govaert, 1992) class that utilizes a hill-climbing approach. EM algorithms initially assign each observation to a cluster and then achieve optimal clustering by alternating between two phases. In the expectation phase, the centers of the different clusters (known as seeds) are computed. The maximization phase involves assigning each observation to its “nearest cluster.” The alternating between the two phases is repeated until no further changes occur in the clusters. Models were constructed for 1 to 8 clusters, and the 4-cluster solution was chosen based on several factors, including the Bayesian information criterion (BIC) (Schwarz, 1978), the elbow method, and ensuring that each cluster had an adequate sample size. The graph showing the BIC per cluster solution is provided in Supplementary Figure S1. The individual trajectories and resulting 4-cluster trajectories are shown in Figure 2A.

**Figure 3:**
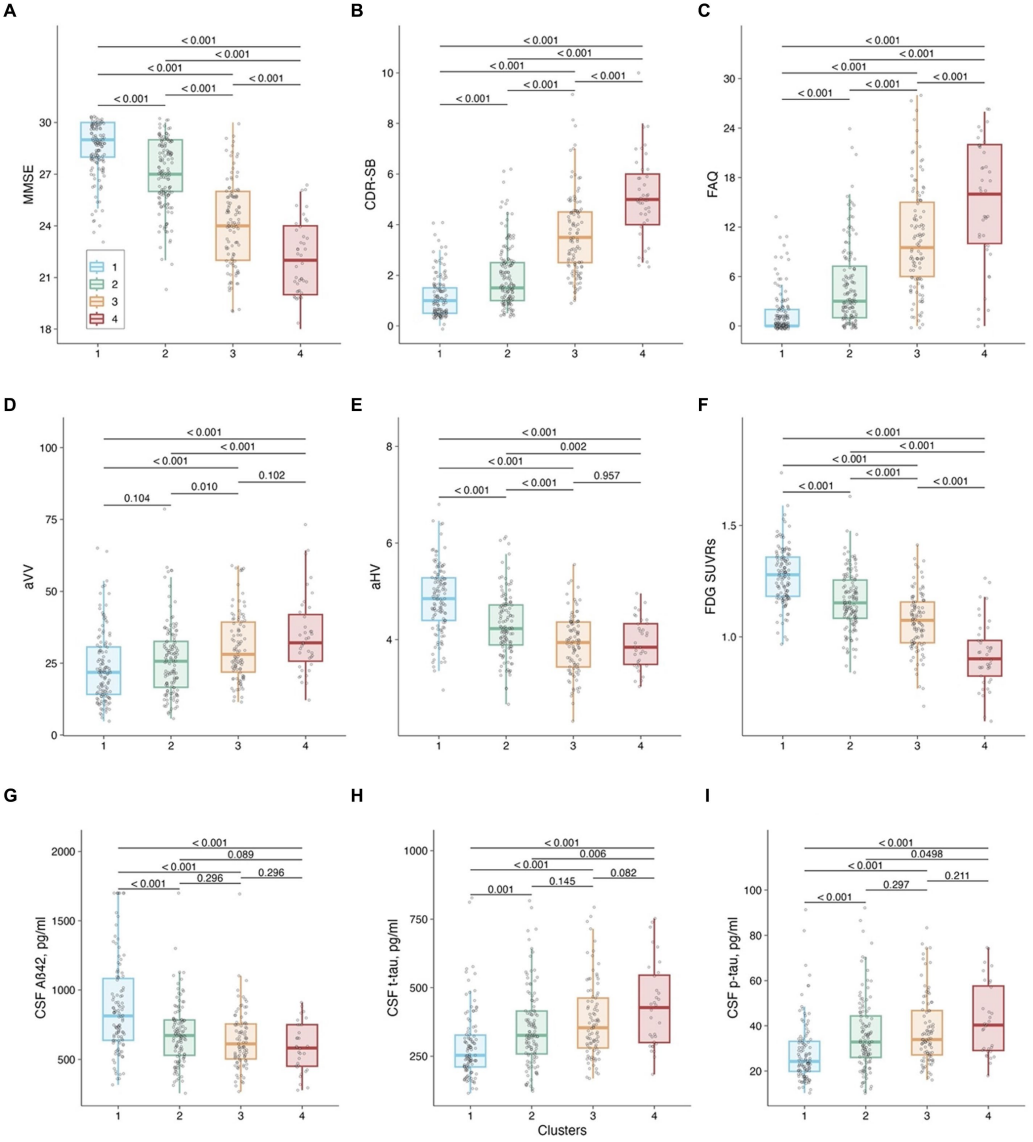
Cluster comparison on baseline cognitive measures, neurogenerative makers, and CSF AD pathologies. Panel **(A)**: MMSE as the outcome. Panel **(B)**: CDR-SB as the outcome. Panel **(C)**: FAQ as the outcome. Panel **(D)**: aVV as the outcome. Panel **(E)**: aHV as the outcome. Panel **(F)**: FDG SUVRs as the outcome. Panel **(G)**: CSF Aβ42 as the outcome. Panel **(H)**: CSF t-tau as the outcome. Panel **(I)**: CSF p-tau as the outcome. Notes: *P* values were adjusted by the FDR method. Abbreviations: MMSE: Mini-Mental State Examination; CDR-SB: Clinical Dementia Rating-sum of boxes; FAQ: Functional Activities Questionnaire; aVV: Adjusted ventricular volume; aHV: Adjusted hippocampal volume; FDG: fludeoxyglucose; Aβ: β-amyloid; t-tau: total tau; p-tau: phosphorylated tau.

Second, the differences in demographics, APOE4 genotype, clinical diagnosis, cognitive evaluations, neurodegenerative markers, and CSF AD biological markers between 4 clusters at baseline were compared. We used analysis of variance (ANOVA) to assess differences between clusters for continuous variables and Pearson’s x^2^ tests for categorical variables. If group differences were detected using ANOVA or Pearson’s x^2^ tests, we performed pairwise t-tests or x^2^ tests in *post hoc* analyses and corrected for multiple testing using the false discovery rate (FDR) correction (Benjamini and Hochberg, 1995). To demonstrate group comparisons of means, we also presented visual representations, as shown in Figure 3.

Third, to characterize the longitudinal changes over a 5-year follow-up period in cognitive measures, neurodegenerative markers, and CSF AD biological markers for each cluster, linear mixed-effects models were built using the R package “lme4” (Bates et al., 2014). We constructed nine models for the dependent variables, including MMSE, CDR-SB, FAQ, aVV, aHV, FDG SUVRs, CSF Aβ42, t-tau, and p-tau. These models included time since baseline (in years), clusters, and their interaction as fixed effects, and age, gender, years of education, APOE4 status, and their interactions with time as covariates. Additionally, a random intercept was included for each participant in all models. Each model was fit using maximum likelihood, which is a method used to estimate the parameters of a statistical model by finding the parameter values that maximize the likelihood function. We also used Satterthwaite’s method to estimate the degrees of freedom in the t-tests, which allowed us to test the significance of the fixed effects and calculate the 95% confidence intervals. The models can be summarized as the following equations: *Y_change_∼ Clusters∗time + Age∗time + Gender∗time + Education∗time +APOE4 status∗time*. *Y_change_* represents the annual change in the aforementioned nine dependent variables from the baseline. To further understand the differences in slopes among the four cluster groups, we conducted pairwise comparisons between clusters using estimated marginal means (EMMs). To adjust for multiple testing, we applied the FDR method for correction.

Fourth, the bootstrap was used to quantify the uncertainty associated with the coefficients and to test the robustness of the results from the aforementioned linear mixed-effects models. We generated 1,000 bootstrapped samples by randomly resampling the data with replacement and refitting the linear mixed-effects models. The random effects were included to generate bootstrapped samples and a semiparametric bootstrap method was performed. The bootstrapped estimates were used to estimate the sampling distribution of the fixed effects, and the 95% confidence intervals (CIs) for the fixed effects were also calculated. A forest plot demonstrating the effect difference relative to Cluster 1 (the reference group) was also created to conduct bootstrap inference.

Fifth, to examine whether cluster membership was predictive of progression from MCI to dementia over a 5-year follow-up period, a Kaplan–Meier plot was utilized to demonstrate the conversion rate to dementia in the four clusters, and log-rank tests were employed to carry out pairwise comparisons of the survival curves and the FDR method was used to correct for multiple testing. Initially, we segregated MCI participants from each cluster, yielding a sample size of 130 in Cluster 1, 119 in Cluster 2, 40 in Cluster 3, and 2 in Cluster 4. Due to the limited number of MCI participants in Cluster 4, we merged MCI participants from Clusters 3 and 4 into a revised Cluster 3. Subsequently, we performed survival analysis utilizing the newly configured cluster variable (consisting of 3 clusters) as the primary predictor. The duration of follow-up was calculated as the interval between the baseline evaluation and the diagnosis of dementia at the last visit. For those subjects who did not develop dementia during the 5-year follow-up period, their data were censored at the time of their last visit.

Sixth, to estimate associations between baseline predictors and cluster membership, multivariable multinomial logistic regression models by the R package “nnet” (Venables and Ripley, 2002) were performed, with Cluster 1 as the reference category. To handle missing values among predictors, 5 complete-data replicates were computed by Multivariate Imputation by Chained Equations using the R package “mice” (Buuren and Groothuis-Oudshoorn, 2011). The overall variable-level missingness is shown in Table 1. We imputed a score using the “predictive mean matching” method, and the imputation model included all predictors that were part of the multinomial logistic regression models, while the outcome variable (cluster membership) was not included in the predictor matrix of the imputation scheme. The multinomial logistic regression modeling was then applied to the 5 imputed data sets and results were pooled utilizing Rubin’s rules to yield estimates and confidence intervals that incorporate the imputed values’ uncertainty (Rubin, 1989). A total of four multinomial logistic regression models with cluster membership as the dependent variable were constructed: the base model included age, gender, years of education, APOE4 genotype, and clinical diagnosis as predictor variables; the cognition model included all the predictors from the base model and additionally incorporated MMSE; the neurodegeneration model further included aHV and FDG-PET at the basis of the cognition model; the AD biomarker model included all the predictors from the neurodegeneration model and additionally incorporated CSF Aβ42 and p-tau proteins. Adjusted odds ratios (ORs) with 95% CIs were computed for all other three clusters by comparing them with Cluster 1 (the reference category).

**Table 1:** Baseline characteristics by cluster.

Characteristic	Overall *N* = 421	Cluster 1 *N* = 131	Cluster 2 *N* = 141	Cluster 3 *N* = 108	Cluster 4 *N* = 41	*p*-value
Age, years	73 (7)	72 (7)	73 (7)	75 (7)^a^	72 (9)^c^	0.035
Education, years	16 (3)	16 (3)	16 (3)	16 (3)	16 (3)	0.12
Female gender, *n* (%)	183 (43%)	59 (45%)	60 (43%)	43 (40%)	21 (51%)	0.6
APOE4 carriers, *n* (%)	287 (68%)	80 (61%)	100 (71%)	77 (71%)	30 (73%)	0.2
Clinical diagnosis, *n* (%)						<0.001
MCI	291 (69%)	130 (99%)	119 (84%)	40 (37%)	2 (4.9%)	
Mild AD	130 (31%)	1 (0.8%)	22 (16%)^a^	68 (63%)^a^^,^^b^	39 (95%)^a^^,^^b^^,^^c^	
Follow-up duration, years	2.55 (1.55)	3.55 (1.37)	2.71 (1.53)^a^	1.66 (1.07)^a^^,^^b^	1.13 (0.66)^a^^,^^b^^,^^c^	<0.001
MMSE	26 (3)	28 (2)	27 (2)^a^	24 (3)^a^^,^^b^	22 (2)^a^^,^^b^^,^^c^	<0.001
CDR-SB	2.45 (1.80)	1.17 (0.84)	2.00 (1.22)^a^	3.57 (1.64)^a^^,^^b^	5.07 (1.68)^a^^,^^b^^,^^c^	<0.001
FAQ	6 (7)	2 (3)	5 (5)^a^	10 (7)^a^^,^^b^	15 (7)^a^^,^^b^^,^^c^	<0.001
Missing, *n*	3	1	1	0	1	
aVV	27 (13)	24 (12)	26 (12)	31 (12)^a^^,^^b^	35 (14)^a^^,^^b^^,^^c^	<0.001
Missing, *n*	28	5	11	10	2	
aHV	4.34 (0.75)	4.84 (0.71)	4.31 (0.67)^a^	3.91 (0.62)^a^^,^^b^	3.92 (0.51)^a^^,^^b^	<0.001
Missing, *n*	43	9	17	12	5	
FDG-PET	1.15 (0.17)	1.28 (0.13)	1.16 (0.13)^a^	1.07 (0.13)^a^^,^^b^	0.92 (0.15)^a^^,^^b^^,^^c^	<0.001
Missing, *n*	8	2	2	4	0	
CSF Aβ42, pg./ml	726 (282)	877 (345)	685 (221)^a^	643 (214)^a^	586 (178)^a^	<0.001
Missing, *n*	74	21	25	19	9	
Aβ positivity based on CSF Aβ42						<0.001
Aβ-	32 (9.2%)	25 (23%)	5 (4.3%)	2 (2.2%)	0 (0%)	
Aβ+	315 (91%)	85 (77%)	111 (96%)^a^	87 (98%)^a^	32 (100%)^a^	
Missing, *n*	74	21	25	19	9	
CSF t-tau, pg./ml	348 (145)	290 (126)	354 (143)^a^	382 (142)^a^	435 (157)^a^^,^^b^	<0.001
Missing, *n*	74	21	25	19	9	
CSF p-tau, pg./ml	35 (16)	28 (14)	36 (16)^a^	38 (15)^a^	43 (16)^a^^,^^b^	<0.001
Missing, *n*	74	21	25	19	9	
AV45 PET	1.41 (0.18)	1.32 (0.16)	1.41 (0.17)^a^	1.46 (0.17)^a^^,^^b^	1.52 (0.16)^a^^,^^b^^,^^c^	< 0.001
Missing, *n*	12	3	6	1	1	
PiB PET	1.91 (0.26)	1.63 (0.18)	2.00 (0.24)	1.95 (NA)	2.06 (0.23)	0.2
Missing, *n*	409	128	135	107	39	

For continuous variables, the mean and standard deviation are used to summarize the data, while for categorical variables, the data are expressed as counts and percentages. We used ANOVA to compare continuous variables and Pearson’s chi-squared tests to compare categorical variables between the four clusters. In case of significant differences detected by ANOVA or Pearson’s chi-squared tests, pairwise t-tests or chi-squared tests were used in post hoc analyses with FDR correction for multiple testing. APOE, Apolipoprotein E; MCI, Mild cognitive impairment; AD, Alzheimer’s disease; MMSE, Mini-Mental State Examination; CDR-SB, Clinical Dementia Rating-sum of boxes; FAQ, Functional Activities Questionnaire; aVV, Adjusted ventricular volume; aHV, Adjusted hippocampal volume; FDG, fludeoxyglucose; Aβ, β-amyloid; t-tau, total tau; p-tau, phosphorylated tau. AV45, Florbetapir; PiB, Pittsburgh Compound B.

a FDR-adjusted *p* < 0.05 compared with Cluster 1.

b FDR-adjusted *p* < 0.05 compared with Cluster 2.

c FDR-adjusted *p* < 0.05 compared with Cluster 3.

Seventh, given that cluster 1 showed negligible decline and cluster 2 only exhibited a slightly higher decline, while clusters 3 and 4 demonstrated similar rates of cognitive decline, we decided to combine clusters 1 and 2 into a group called “non/slow decliners,” and merge clusters 3 and 4 into a group referred to as “steep decliners.” Binary logistic regression models were built with same predictors included in the AD biomarker model and the two groups (non/slow decliners vs. steep decliners) as the outcome. To select the predictors that explain the bulk of variance in cognitive decline and create a simplified model for practicality, we employed a fast backward variable selection method (Lawless and Singhal, 1978), using the total residual Akaike’s information criterion (AIC) (Akaike, 1974) as the stopping rule. A nomogram was created to facilitate an easy and visual estimation of probabilities of steep cognitive decline based on the reduced model. Finally, in order to examine whether the inclusion of our nomogram for enrichment can reduce sample size, simulation of clinical trials were performed using the longpower R package, with a 25% treatment effect on cognitive performance over time, a statistical power of 80%, 1:1 allocation of placebo and treatment groups, and a total duration of 18 months with cognitive assessments at 0, 3, 6, 9, 12, 15, and 18 months. Bootstrap with 500 iterations was conducted.

## Results

### Findings of longitudinal cluster analysis

Four clusters were identified. Figure 2A demonstrates the categorization of participants with symptomatic early AD based on their ADAS-Cog-13 trajectories over a 5-year follow-up period into the following clusters: Cluster 1 (*n* = 131, 31%), characterized by stable cognitive performance; Cluster 2 (*n* = 141, 34%), characterized by a mild cognitive decline; Clusters 3 (*n* = 108, 26%) and 4 (*n* = 41, 9%), characterized by moderate to rapid rates of cognitive decline, with Cluster 4 showing more impaired cognitive performance at baseline and greater rates of cognitive decline than Cluster 3 as shown by the intercepts and slopes.

### Baseline cluster characteristics

As shown in Table 1 and Figures 2B–D, 3, the baseline sociodemographic and clinical characteristics were compared between clusters. For age, Cluster 3 was older than Cluster 1, and Cluster 4 was younger than Cluster 3, but no other pairwise difference was observed. For years of education, APOE4 status (Figure 2B), and gender (Figure 2C), clusters did not differ significantly. For clinical diagnosis (Figure 2D), the distribution of mild AD dementia was significantly different between each cluster, with Cluster 4 having the highest percentage of patients with mild AD dementia. For follow-up duration, four clusters differed significantly, with Cluster 1 having the longest follow-up duration. Regarding cognitive and functional measures, all tests (MMSE, CDR-SB, and FAQ) showed significant differences between the four clusters. For neurodegenerative markers (aVV, aHV, and FDG-PET), four clusters showed significant differences, except no difference in aVV between clusters 1 and 2 and no difference in aHV between clusters 3 and 4. Regarding CSF Aβ42 levels, Clusters 2–4 had lower levels of CSF Aβ42 than Cluster 1, while no other pairwise difference was observed. Regarding CSF tau proteins, Clusters 2–4 had higher levels of CSF t-tau and p-tau than Cluster 1, and Cluster 4 had higher levels of CSF t-tau and p-tau than Cluster 2, while no other pairwise difference was observed.

### Associations of cluster membership with longitudinal changes in cognitive measures, neurodegenerative markers, and CSF AD biological markers

**Figure 4:**
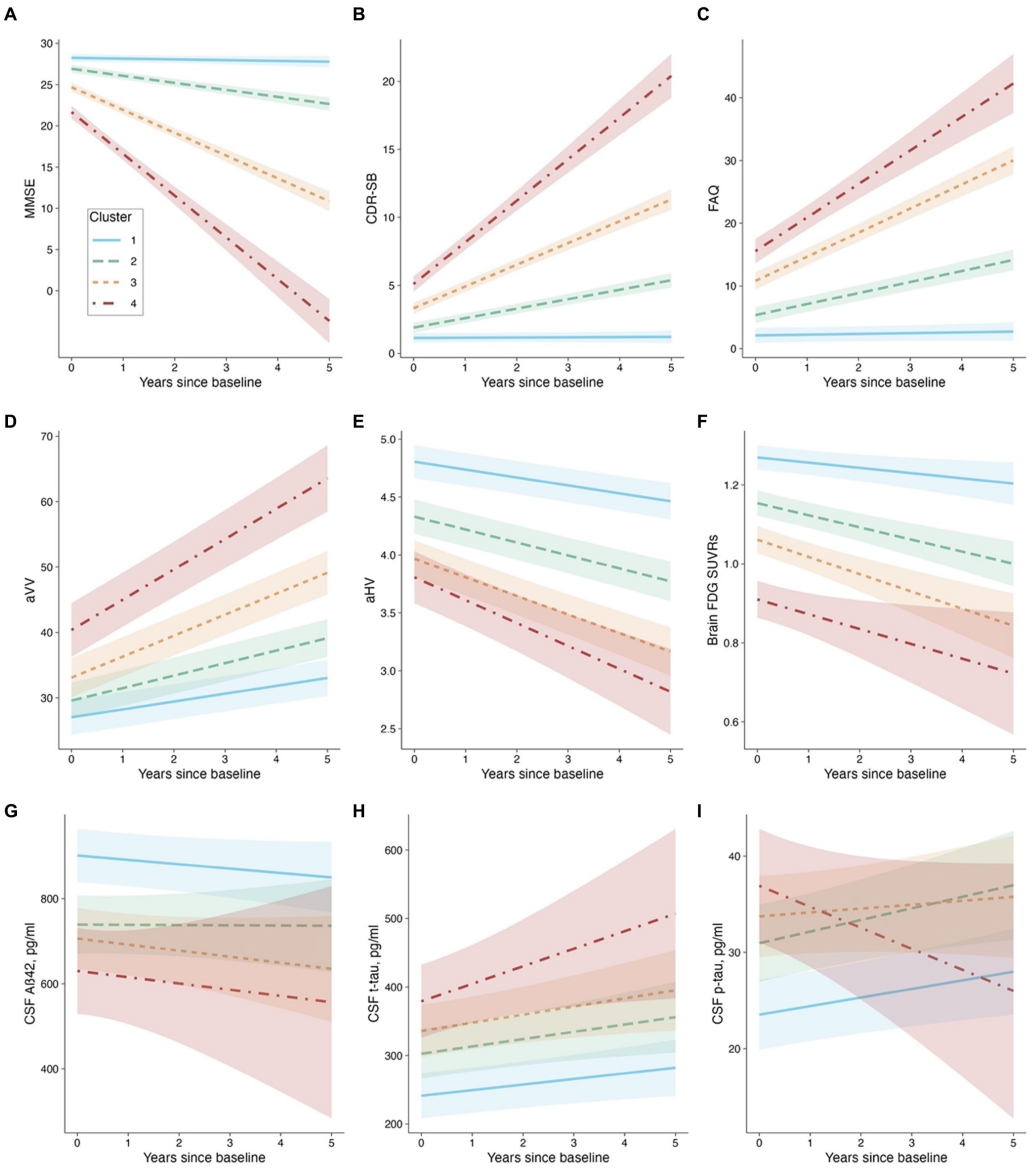
Cluster membership and longitudinal changes. Panel **(A)**: MMSE as the outcome. Panel **(B)**: CDR-SB as the outcome. Panel **(C)**: FAQ as the outcome. Panel **(D)**: aVV as the outcome. Panel **(E)**: aHV as the outcome. Panel **(F)**: FDG SUVRs as the outcome. Panel **(G)**: CSF Aβ42 as the outcome. Panel **(H)**: CSF t-tau as the outcome. Panel **(I)**: CSF p-tau as the outcome. Abbreviations: MMSE: Mini-Mental State Examination; CDR-SB: Clinical Dementia Rating-sum of boxes; FAQ: Functional Activities Questionnaire; aVV: Adjusted ventricular volume; aHV: Adjusted hippocampal volume; FDG: fludeoxyglucose; Aβ: β-amyloid, t-tau: total tau, p-tau: phosphorylated tau.

The results of the linear mixed-effects models, which investigated the relationship between cluster membership and longitudinal changes in other cognitive measures, neurodegenerative markers, and CSF AD pathologies over a 5-year follow-up period, are presented in Table 2 and Figure 4.

**Table 2:** Summary of linear mixed effects models.

Predictors	Coefficients	SE	*p*-value	Coefficients	SE	*p*-value	Coefficients	SE	*p*-value
	MMSE			CDR-SB			FAQ		
Age × time	0.00517	0.00543	0.34103	0.00501	0.00318	0.115	−0.00853	0.00906	0.3468
Female gender × time	−0.03209	0.07337	0.66196	0.04473	0.04344	0.303	0.14227	0.12266	0.2463
Education × time	−0.00308	0.01374	0.82277	−0.00768	0.00818	0.348	−0.02221	0.02299	0.3342
APOE4 carriers × time	−0.09874	0.07737	0.20211	0.07859	0.04563	0.085	0.34530	0.12932	0.0077
Cluster 2 × time	−0.76090	0.07896	< 0.001	0.68107	0.04656	< 0.001	1.63580	0.13178	< 0.001
Cluster 3 × time	−2.67502	0.12525	< 0.001	1.58459	0.07449	< 0.001	3.72023	0.20776	< 0.001
Cluster 4 × time	−4.97333	0.29352	< 0.001	3.04552	0.17219	< 0.001	5.22412	0.49006	< 0.001
	aVV			aHV			FDG SUVRs		
Age × time	0.00450	0.00739	0.543	−0.00186	0.00068	0.0059	0.00005	0.00038	0.89570
Female gender × time	−0.09106	0.09402	0.333	−0.02882	0.00889	0.0012	−0.00857	0.00499	0.08743
Education × time	0.01274	0.01694	0.452	0.00307	0.00167	0.0667	−0.00009	0.00087	0.91645
APOE4 carriers × time	0.19189	0.09692	0.048	−0.01589	0.00944	0.0926	−0.00242	0.00500	0.62848
Cluster 2 × time	0.70912	0.09689	< 0.001	−0.04347	0.00949	< 0.001	−0.01746	0.00505	< 0.001
Cluster 3 × time	2.00695	0.16203	< 0.001	−0.09211	0.01549	< 0.001	−0.03043	0.00756	< 0.001
Cluster 4 × time	3.43357	0.32123	< 0.001	−0.12947	0.03251	< 0.001	−0.02434	0.01518	0.11045
	CSF Aβ42			CSF t-tau			CSF p-tau		
Age × time	−0.0573	0.5792	0.9213	0.168	0.251	0.50419	−0.0212	0.0270	0.43234
Female gender × time	−5.7159	7.7435	0.4613	0.346	3.356	0.91793	−0.1093	0.3603	0.76202
Education × time	−0.8924	1.3041	0.4946	−0.579	0.571	0.31207	−0.0185	0.0607	0.76075
APOE4 carriers × time	−9.3435	8.0439	0.2468	−5.199	3.493	0.13829	−0.5993	0.3741	0.11084
Cluster 2 × time	9.7768	8.3837	0.2449	2.498	3.613	0.49024	0.3131	0.3897	0.42282
Cluster 3 × time	−3.9152	11.1462	0.7258	3.690	4.865	0.44909	−0.4855	0.5194	0.35108
Cluster 4 × time	−4.3862	25.9948	0.8662	17.413	11.405	0.12841	−3.0757	1.2123	0.01196

All models include the main effects of predictors, such as age, gender, years of education, APOE4 status, cluster membership, and years since baseline. However, for the sake of brevity, their coefficients are not displayed. Coefficients represent unstandardized values that indicate the yearly changes in each AD biomarker. APOE, Apolipoprotein E; MMSE, Mini-Mental State Examination; CDR-SB, Clinical Dementia Rating-sum of boxes; FAQ, Functional Activities Questionnaire; aVV, Adjusted ventricular volume; aHV, Adjusted hippocampal volume; FDG, fludeoxyglucose; Aβ, β-amyloid; t-tau, total tau; p-tau, phosphorylated tau.

Concerning the models that involve cognitive and functional assessments (MMSE, CDR-SB, and FAQ), our analysis revealed that Clusters 2 through 4 experienced significantly greater decline (or worsening) relative to Cluster 1 (refer to Table 2 and Figures 4A–C). Utilizing EMMs for pairwise comparisons, we observed that the differences in slopes were statistically significant between each cluster (with all FDR-adjusted *p*-values being less than 0.0001).

For the aVV model (see Table 2 and Figure 4D), the cluster × time interactions were all significant, indicating that Clusters 2–4 had greater slopes (i.e., faster rates of ventricular enlargement) compared to Cluster 1. Pairwise comparisons showed that the slope difference was significant between each cluster (all FDR-adjusted *p* < 0.0001). For the aHV model (see Table 2; Figure 4E), the cluster × time interactions were all significant, indicating that Clusters 2–4 had steeper slopes (i.e., faster rates of hippocampal atrophy) compared to Cluster 1. Pairwise comparisons showed that the slope difference was significant between each cluster (all FDR-adjusted *p* < 0.05), except for a comparable level between Clusters 3 and 4 (FDR-adjusted *p* = 0.289). For the FDG-PET model (see Table 2 and Figure 4F), Clusters 2 and 3, but not Cluster 4, had steeper slopes (i.e., faster decline in brain glucose metabolism) compared to Cluster 1. Pairwise comparisons showed that the slope difference was significant between Clusters 1 and 2 (FDR-adjusted *p* = 0.0026), and between Clusters 1 and 3 (FDR-adjusted *p* = 0.007), while no other pairwise comparison was significant (all FDR-adjusted *p* > 0.05).

Regarding the models that invovle CSF AD biological markers (CSF Aβ42, t-tau, and p-tau levels), the cluster × time interactions were not significant, except for a slope difference on CSF p-tau between Clusters 4 and 1 (see Table 2 and Figures 4G–I). These findings suggested that the changes in CSF AD biomarkers over time were consistent across clusters, with the exception of CSF p-tau, where a difference in the rate of change was observed between Clusters 4 and 1. Pairwise comparisons showed that there were significant slope differences in CSF p-tau between Clusters 4 and 1 (FDR-adjusted *p* = 0.04), and between Clusters 4 and 2 (FDR-adjusted *p* = 0.04), while no other pairwise comparison was significant (all FDR-adjusted *p* > 0.05).

Additionally, a semiparametric bootstrap method was performed to quantify the uncertainty associated with the coefficients and to test the robustness of the results from the linear mixed-effects models (Supplementary Figure S2). For example, as shown in Supplementary Figures S2A–C, the findings from the models involving cognitive and functional measures can further be evidenced by visually examining the forest plots showing the slope difference relative to Cluster 1 (the reference group). The plots demonstrate how much the effect of interest in each of the three clusters differed relative to Cluster 1. All effects were significant relative to the reference group because none of the 95% confidence intervals contained 0. Additionally, the coefficients of Clusters 2–4 fell outside each other’s intervals, indicating that they were significantly different from each other. For other models, the results were largely unchanged and consistent with the pairwise comparisons described above.

### Clinical progression from MCI to dementia

**Figure 5:**
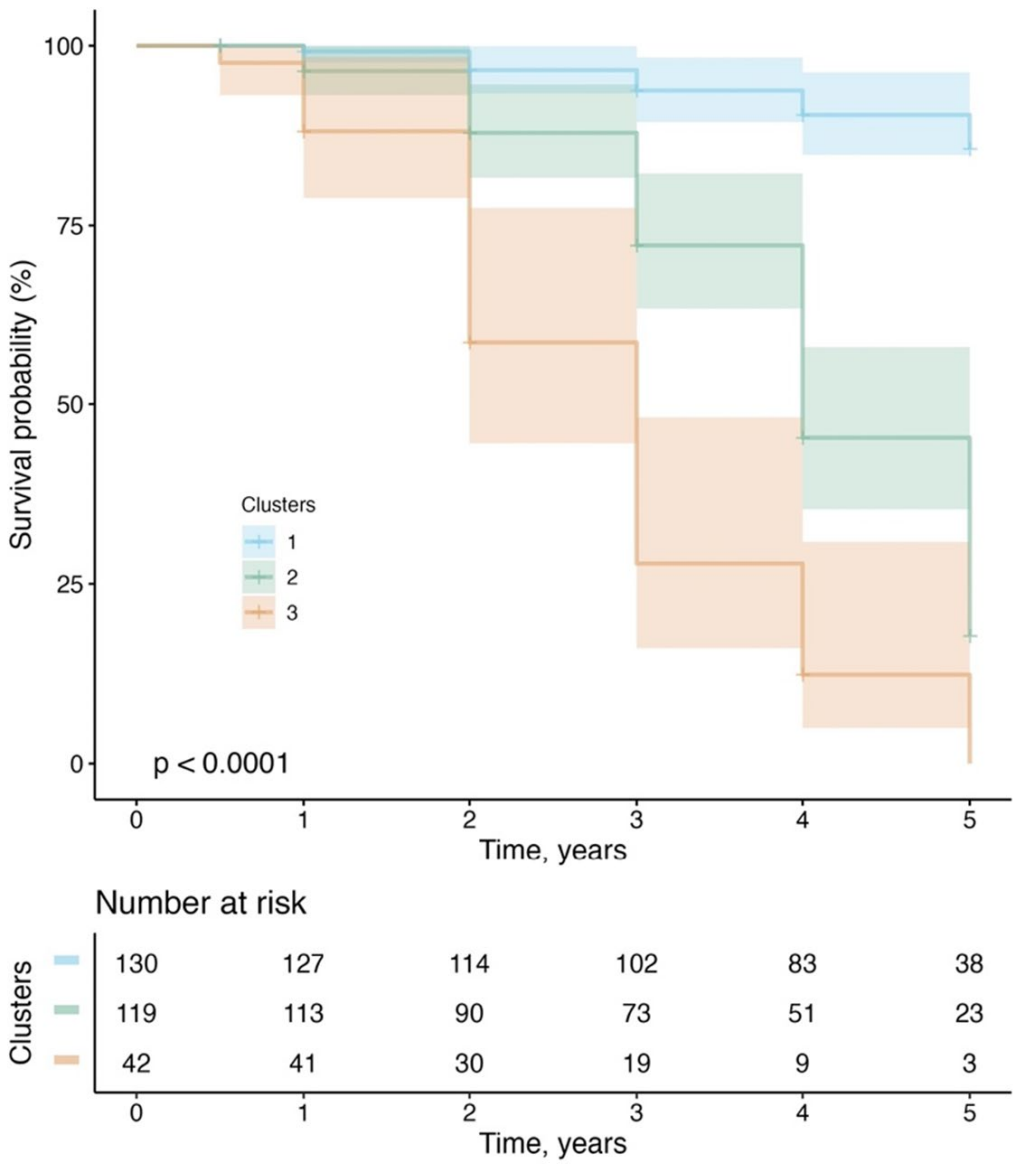
Progression from MCI to dementia.

Of the 291 MCI participants, two participants initially assigned to Cluster 4 in the cluster analysis (see Table 1) were reclassified as Cluster 3 due to the inadequacy of their small size as an independent group. Therefore, the cluster variable used in the survival analysis included 3 subgroups rather than 4 subgroups. Over a 5-year follow-up period, 103 (35%) participants converted to dementia. Figure 5 demonstrates the conversion rate to dementia using the Kaplan–Meier curves. A significant cluster difference in the conversion rate was observed using a log-rank test (x^2^[2] = 124; *p* < 0.001). All pairwise comparisons between the 3 clusters were significant (all FDR-adjusted *p* < 0.001). With regard to the type of dementia, of the 103 MCI participants who progressed to dementia, 98 (95.1%) participants were diagnosed with AD dementia, while 5 (4.85%) progressed to non-AD dementias, including 2 with primary progressive aphasia, 1 with Parkinson’s disease and Lewy body dementia features, 1 with progressive supranuclear palsy, and 1 who had experienced delirium in the past after being infected with the West Nile Virus.

### Findings of multinomial logistic regression models

Multinomial logistic regression models were performed to examine the associations between potential predictors at baseline and cluster membership. Table 3 shows the summary of multinomial logistic regression models. We built four models and assessed model fit using Nagelkerke’s R^2^ (Nagelkerke, 1991). In the base model, all predictors captured 47.5% of the variability in the Cluster outcome (R^2^ = 0.475). The cognition model explained 56.7% of the variability in the Cluster outcome (R^2^ = 0.567), indicating that the MMSE explained an additional 9.2% of the total Cluster variability. In the neurodegeneration model, all predictors captured 67.4% of the variability in the Cluster outcome (R^2^ = 0.674), suggesting that aHV and FDG-PET explained an additional 10.7% of the total Cluster variability. Meanwhile, the AD biomarker model explained 68.8% of the variability in the Cluster outcome (R^2^ = 0.688), indicating that CSF Aβ42 and p-tau explained only an additional 1.4% of the total Cluster variability. Table 3 displays the adjusted odds ratios (aOR) and their 95% confidence intervals (CIs) for each model. For instance, in the AD biomarker model (the full model), compared with Cluster 1, there were consistent associations of membership to Cluster 2–4 with a clinical diagnosis of AD (aOR [95% CI]: 5.41 [2.54, 11.5], 12.6 [5.85, 27.1], and 44.9 [16.6, 121], respectively). MMSE, aHV, FDG-PET, and CSF p-tau were also consistently associated with cluster membership to Cluster 2–4. However, there were no significant associations between CSF Aβ42 levels and cluster membership.

**Table 3:** Summary of multinomial logistic regression models.

	Clusters 2 *vs* 1		Clusters 3 *vs* 1		Clusters 4 *vs* 1	
Predictors	aOR (95% CI)	*p*-value	aOR (95%CI)	*p*-value	aOR (95%CI)	*p*-value
*The base model*						
Age, continuous (1-year increment)	1.03 (0.99–1.07)	0.2	1.04 (1.00, 1.10)	0.076	0.99 (0.93, 1.06)	0.8
Female gender, categorical (male as reference)	0.92 (0.55–1.53)	0.7	0.85 (0.44, 1.64)	0.6	1.37 (0.54, 3.51)	0.5
Education, continuous (1-year increment)	0.95 (0.86–1.04)	0.3	0.92 (0.81, 1.03)	0.15	0.96 (0.80, 1.14)	0.6
APOE4 carriers, categorical (noncarriers as reference)	1.70 (1.00–2.89)	0.050	1.84 (0.92, 3.68)	0.084	1.84 (0.67, 5.04)	0.2
Clinical diagnosis, categorical (MCI as reference)	23.8 (3.13–181)	0.002	215 (28.5, 1,613)	< 0.001	2,560 (223, 29,330)	< 0.001
*The cognition model*						
Age, continuous (1-year increment)	1.02 (0.98, 1.06)	0.4	1.03 (0.97, 1.08)	0.3	0.97 (0.91, 1.04)	0.3
Female gender, categorical (male as reference)	0.97 (0.57, 1.65)	> 0.9	0.97 (0.47, 1.99)	> 0.9	1.95 (0.69, 5.49)	0.2
Education, continuous (1-year increment)	0.99 (0.90, 1.09)	0.8	1.00 (0.87, 1.14)	> 0.9	1.08 (0.89, 1.32)	0.4
APOE4 carriers, categorical (noncarriers as reference)	1.57 (0.91, 2.71)	0.11	1.54 (0.73, 3.26)	0.3	1.73 (0.57, 5.22)	0.3
Clinical diagnosis, categorical (MCI as reference)	7.86 (0.97, 63.7)	0.054	23.4 (2.88, 189)	0.003	105 (8.2, 1,352)	< 0.001
MMSE, continuous (1-unit increment)	0.71 (0.60, 0.84)	< 0.001	0.49 (0.40, 0.61)	< 0.001	0.35 (0.26, 0.47)	< 0.001
*The neurodegeneration model*						
Age, continuous (1-year increment)	0.97 (0.93, 1.02)	0.3	0.97 (0.91, 1.03)	0.4	0.96 (0.88, 1.04)	0.3
Female gender, categorical (male as reference)	1.34 (0.74, 2.44)	0.3	1.6 (0.71, 3.61)	0.3	3.94 (1.21, 12.8)	0.023
Education, continuous (1-year increment)	0.95 (0.85, 1.06)	0.3	0.93 (0.81, 1.08)	0.4	0.99 (0.79, 1.25)	> 0.9
APOE4 carriers, categorical (noncarriers as reference)	1.27 (0.68, 2.35)	0.5	1.34 (0.58, 3.10)	0.5	1.86 (0.53, 6.52)	0.3
Clinical diagnosis, categorical (MCI as reference)	5.14 (0.6, 44.2)	0.14	12 (1.35, 107)	0.026	40.4 (2.69, 605)	0.008
MMSE, continuous (1-unit increment)	0.73 (0.61, 0.88)	< 0.001	0.52 (0.41, 0.66)	< 0.001	0.4 (0.29, 0.55)	< 0.001
aHV, continuous (1-unit increment)	0.43 (0.25, 0.72)	0.002	0.23 (0.11, 0.47)	< 0.001	0.28 (0.10, 0.82)	0.020
FDG-PET, continuous (1-unit increment)	0.01 (0.00, 0.06)	< 0.001	0.00 (0.00, 0.01)	< 0.001	0.00 (0.00, 0.00)	< 0.001
*The AD biomarker model*						
Age, continuous (1-year increment)	0.97 (0.94, 1.01)	0.12	0.97 (0.92, 1.02)	0.2	0.96 (0.90, 1.02)	0.2
Female gender, categorical (male as reference)	1.25 (0.66, 2.36)	0.5	1.39 (0.59, 3.28)	0.5	3.45 (0.98, 12.2)	0.054
Education, continuous (1-year increment)	0.96 (0.86, 1.08)	0.5	0.95 (0.82, 1.10)	0.5	0.99 (0.80, 1.23)	> 0.9
APOE4 carriers, categorical (noncarriers as reference)	0.91 (0.47, 1.76)	0.8	0.93 (0.39, 2.23)	0.9	1.14 (0.30, 4.30)	0.9
Clinical diagnosis, categorical (MCI as reference)	5.41 (2.54, 11.5)	< 0.001	12.6 (5.85, 27.1)	< 0.001	44.9 (16.6, 121)	< 0.001
MMSE, continuous (1-unit increment)	0.75 (0.66, 0.86)	< 0.001	0.54 (0.45, 0.64)	< 0.001	0.42 (0.32, 0.55)	< 0.001
aHV, continuous (1-unit increment)	0.42 (0.26, 0.69)	< 0.001	0.21 (0.11, 0.40)	< 0.001	0.23 (0.09, 0.60)	0.003
FDG-PET, continuous (1-unit increment)	0.01 (0.00, 0.05)	< 0.001	0.00 (0.00, 0.00)	< 0.001	0.00 (0.00, 0.00)	< 0.001
CSF Aβ42 levels, continuous (1-unit increment)	1.00 (1.00, 1.00)	0.051	1.00 (1.00, 1.00)	0.093	1.00 (0.99, 1.00)	0.15
CSF p-tau levels, continuous (1-unit increment)	1.02 (1.00, 1.05)	0.043	1.04 (1.01, 1.07)	0.019	1.06 (1.01, 1.1,)	0.019

aOR, adjusted odds ratio; CI, confidence interval; APOE, Apolipoprotein E; MMSE, Mini-Mental State Examination; aHV, Adjusted hippocampal volume; FDG, fludeoxyglucose; Aβ, β-amyloid; p-tau, phosphorylated tau.

### A simpler predictive model for enrichment of clinical trials involving early AD

**Figure 6:**
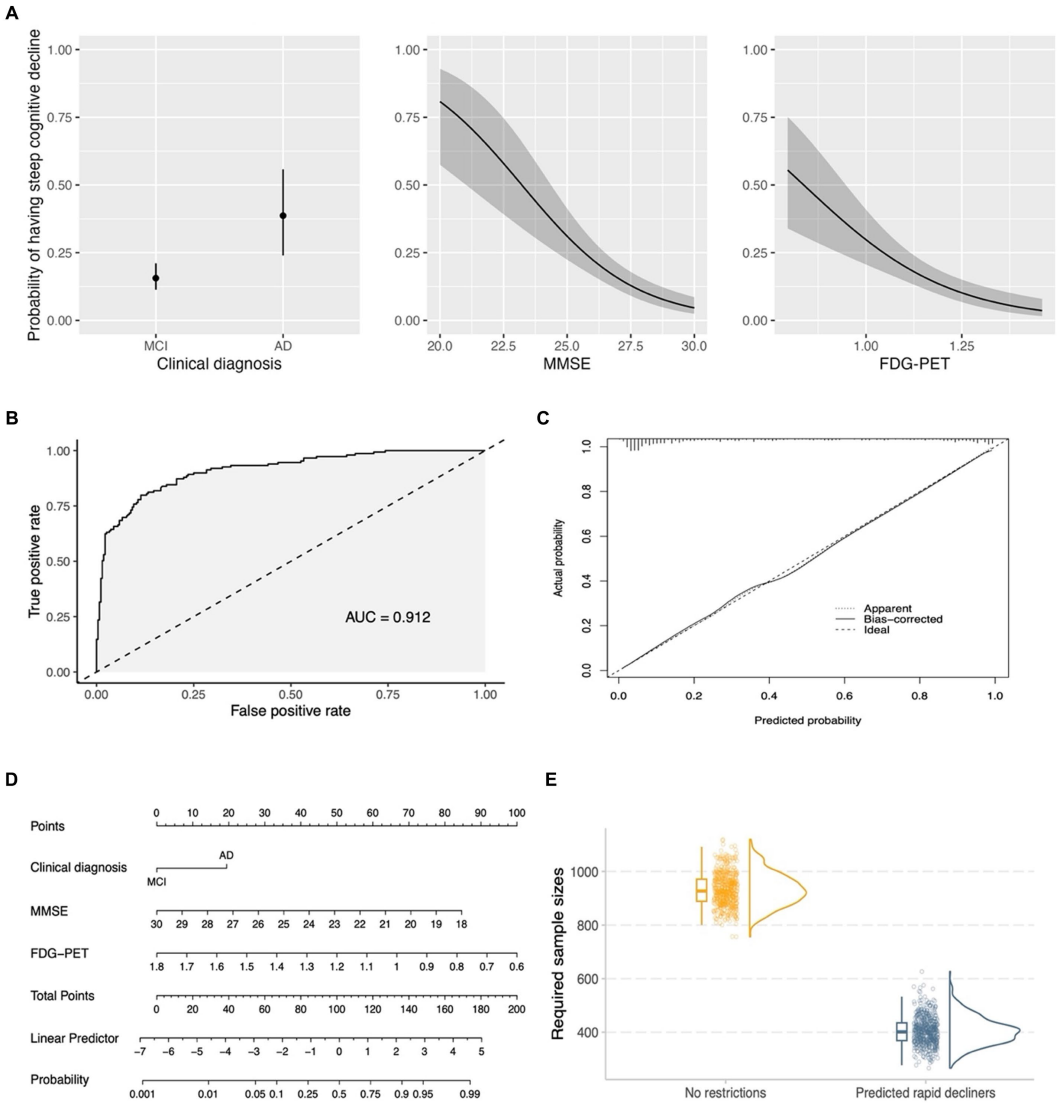
A simplified model for enrichment of clinical trials involving early AD. **(A)** Shows the partial effects of three predictors in the simplified model. The partial effect of a predictor was investigated when holding all other predictors constant (clinical diagnosis = MCI; median MMSE score = 27; median FDG-PET = 1.158). **(B)** Demonstrates the ROC curve for the simplified model (clinical diagnosis, MMSE score, and FDG-PET). **(C)** Shows a bootstrap overfitting-corrected calibration curve. The closeness of the calibration curve to the 45° line illustrates an excellent calibration on the probability scale. **(D)** Displays a nomogram for the prediction of probabilities of experiencing steep cognitive decline over time based on 3 predictors in the simplified model. Instructions for using the nomogram: start by identifying the individual’s clinical diagnosis (MCI or mild AD) and draw a vertical line to intersect on the Points axis (i.e., the top axis) to find the point. Repeat this process for the other predictors (MMSE and FDG-PET). Add up the points obtained for these three predictors. Locate the final sum on the Total Points scale and draw a vertical line downwards to identify the individual’s predicted probability of experiencing steep cognitive decline on the Probability axis (i.e., the bottom axis). **(E)** Shows that the usage of the nomogram for enrichment resulted in a substantial reduction of required sample sizes in simulated clinical trials of early AD. For each column of the **(E)** (no restrictions vs. Predicted rapid decliners), three different plots are presented, including a boxplot, raw data points, and a density plot. MCI, Mild cognitive impairment; AD, Alzheimer’s disease; MMSE, Mini-Mental State Examination; FDG, fludeoxyglucose; AUC, Area Under the Curve.

Model simplification was conducted using stepwise variable selection with AIC as the stopping rule (see the seventh point in the statistical analyses section). The variables screened for inclusion were the same predictors as those in the AD biomarker model (see Table 3). The reduced model included clinical diagnosis, MMSE score, and FDG-PET, and partial effects plots with 95% pointwise confidence bands are presented in Figure 6A. This model resulted in an Area Under the Curve (AUC) of 0.912 (95% confidence intervals: 0.88–0.94; using DeLong statistics; Figure 6B). To evaluate the reliability of the model, a bootstrap overfitting-corrected calibration curve was generated with 1,000 iterations. Figure 6C demonstrates the excellent calibration of the model on the probability scale, as indicated by the close alignment of the calibration curve with the 45° line. Internal validation was performed using the bootstrap technique with 1,000 iterations, resulting in an optimism-corrected AUC of 0.910. A nomogram was created to facilitate an easy and practical estimation of the probabilities of experiencing steep cognitive decline in early AD (Figure 6D). Finally, simulated clinical trials were performed to investigate whether the nomogram would be used to enrich for trial populations in order to reduce sample size for a clinical trial involving individuals with early AD. As shown in Figure 6E, when including all eligible patients (no restrictions), the required sample sizes were 926.8 (95% CI: 822.6–1057.5). However, when including individuals predicted to experience steep cognitive decline using the nomogram, the required sample sizes were decreased to 400.9 (95% CI: 306.9–516.8).

## Discussion

In this study, we observed highly variable cognitive trajectories in patients with early AD with evidence of abnormal amyloid in the brain already and demonstrated that AD does not affect different individuals in a uniform manner. In addition, our clustering analysis categorized patients with early AD into four subgroups and visualized these individual differences in patterns of cognitive trajectories. Overall, the findings of the current study provide evidence of (1) heterogeneous patterns of cognitive decline between patients with early AD, (2) associations of cognitive heterogeneity with other commonly used cognitive and functional measures, neurodegenerative markers, CSF AD biomarkers, and clinical progression from the MCI stage to a dementia diagnosis, (3) relationships of several baseline predictors with membership in the identified subtypes, and (4) the clinical relevance of a nomogram in predicting the probabilities of experiencing steep cognitive decline based on the simplified model.

Our data-driven clustering method successfully categorized patients with early AD into biologically and clinically different subgroups, as evidenced by the substantial differences in clinical characteristics and longitudinal changes in major AD biomarkers among the four identified clusters. Cluster 1, which represented approximately 31% of our study sample, showed a nearly negligible rate of change in the ADAS-Cog-13 over a 5-year follow-up period (Figure 2A). This subgroup had significantly better baseline performance on other cognitive and functional measures, including the MMSE, CDR-SB, and FAQ, as well as a more favorable biomarker profile. These findings are consistent with previous studies conducted in clinical trial-based samples (Geifman et al., 2017; Levine et al., 2021) and the ADNI dataset (Ziegler et al., 2020), which also identified a similar subgroup with less impaired baseline performance. More importantly, CSF Aβ42 levels of a number of individuals in cluster 1 were well above the level that would be regarded CSF Aβ42 positive. In addition, cluster 1 was also experiencing minimal or no change in CSF Aβ42 over time, further indicating that these individuals might not be on the AD trajectory. One reasonable strategy to maximize the chances of finding a successful treatment is to identify and remove the subgroup with a very low rate of cognitive decline in clinical trials (Edmonds et al., 2018). Therefore, improved stratification of early AD populations prior to recruitment in clinical trials could enhance the likelihood of detecting treatment efficacy and aid in the development of more efficient study designs.

We identified the largest early AD subgroup of the study sample, Cluster 2 (*n* = 141, 34%), which initially performed worse on the ADAS-Cog-13, as shown by a greater intercept in Figure 2A, and exhibited a slightly faster rate of cognitive deterioration over time than Cluster 1. The two subgroups (Clusters 1 and 2) also differed significantly in their levels of AD-associated biomarkers, as indicated by Figures 3G–I. Specifically, participants in Cluster 2 had higher levels of CSF tau pathologies and lower levels of CSF Aβ42 at baseline compared to those in Cluster 1. These findings are consistent with several longitudinal studies showing that unfavorable CSF biomarker profiles at baseline are predictive of future cognitive decline in patients with very mild AD dementia or in healthy older adults (Snider et al., 2009; Stomrud et al., 2010). Neurodegenerative markers, including aHV and FDG SUVRs (Figures 3E,F), were also significantly different between the two cognitive trajectories, suggesting that higher levels of aHV and FDG SUVRs (indicative of less severe neurodegeneration at baseline) are associated with slower cognitive deterioration over time in patients with early AD (de Leon et al., 2001; Lo et al., 2011). However, aVV did not show a significant difference between the two clusters (Figure 3D) despite that all three neuroimaging markers are considered indicators of neurodegeneration. These discrepancies may be attributed to the fact that ventricular enlargement is considered a more downstream event that is closely coupled with overt cognitive decline in the AD pathophysiological changes (Jack et al., 2004). Therefore, aVV may be a less sensitive marker at baseline compared to aHV and FDG SUVRs to predict future cognitive decline and may not be able to capture the subtle differences in cognitive decline between Clusters 1 and 2.

Clusters 3 (*n* = 108, 26%) and 4 (*n* = 41, 9%) demonstrated significantly steeper cognitive deterioration over time than Clusters 1 and 2, with Cluster 4 exhibiting the fastest cognitive decline trajectory (Figure 2A). These findings have important implications for clinical trials involving patients with early AD. In a clinical trial, assuming that the rates of cognitive decline exceed the level of background noise, randomization procedures may not necessarily lead to equal rates of decline in cognitive function between the treatment and placebo groups (Deaton and Cartwright, 2016). As a consequence, the variation in cognitive decline may affect the result of a trial. For instance, although a statistically significant difference was found between the placebo and treatment groups in previous clinical trials, it cannot be entirely ruled out that the group difference may have been due to an over-representation of individuals from Clusters 3 and 4 (fast cognitive decliners) in the placebo group, or an over-representation of individuals from Clusters 1 and 2 (slow cognitive decliners) in the treatment group. One approach to overcome the problem of cognitive heterogeneity might thus be to recruit trial participants based on risk factors that are able to predict cognitive trajectories they may fall into subsequently. Future trials may consider enrolling individuals predicted to fall into Clusters 3 and 4 in order to increase the chances of success, enhance statistical power to observe treatment effects, and reduce cost, duration, and required sample size of a trial. One of the first steps to testing this strategy might be to build a multinomial logistic regression model to predict the cluster membership using baseline AD-associated markers. Our findings, as shown in Table 3, illustrated that in patients with early AD (with evidence of abnormal amyloid), clinical status, MMSE scores, aHV, FDG SUVRs, and CSF p-tau levels, which are well known factors related with rate of clinical progression (Blanco et al., 2023), were associated with the cluster membership. For instance, the AD biomarker model can explain 68.8% of the variability of the cluster membership. However, we did not observe a significant association between CSF Aβ42 levels and the membership of cognitive trajectories in patients with early AD. This finding is somewhat expected and is probably explained by the fact that all included individuals were already amyloid-positive, and the variations in CSF Aβ42 levels were not large enough to effectively capture the cognitive change. In addition, amyloid accumulation is thought of as a very early event that occurs prior to hypometabolism, hippocampal atrophy, or cognitive decline and has an early and subclinical effect on cognition (Lo et al., 2011). The simplified binary logistic regression model (Figure 6) may serve as the first crucial step in enrolling “right” participants (Cummings et al., 2019) and powering clinical trial design. Our model offers a valuable tool for the design and execution of new clinical trials. By complementing standard inclusion/exclusion criteria, they serve as an enhancement strategy to curate a cohort enriched with participants who are forecasted to exhibit cognitive decline. This approach simultaneously safeguards against the inadvertent enrollment of individuals anticipated to maintain cognitive stability, ensuring a more focused and efficient study population. Future studies that focus on replicating and validating our results in an independent cohort and testing whether our model is useful in the enrichment of clinical trials in early AD by identifying participants with faster cognitive trajectories are warranted.

The temporal ordering of changes in AD biomarkers can provide crucial information for our understanding of the pathophysiological alterations of the disease and for designing clinical trials for AD. The hypothetical cascade of AD biomarker changes has been proposed to follow a specific temporal ordering from amyloid deposition to tau aggregation, then to brain metabolic and structural changes, and finally to cognitive symptoms (Jack et al., 2013). However, this model remains to be tested. Our findings from these models largely support the hypothetical orderings of biomarker changes (Jack et al., 2013). Our analyses suggested that rates of cognitive progression were loosely coupled or even not associated with changes in markers presumed to be upstream events (i.e., changes in CSF Aβ42 and tau proteins) while were relatively more coupled with changes in markers presumed to indicate neurodegeneration and neuronal injury (i.e., ventricular enlargement, hippocampal atrophy, and brain hypometabolism). Specifically, our analyses did not observe a significant difference in the rates of change in CSF Aβ42 or tau proteins between four cognitive trajectories (Figures 4G,H; Supplementary Figures S2G,H), indicating that amyloid accumulation or tau aggregation (CSF-based measures) may have reached a plateau at the MCI and mild dementia stages of the disease. The negative slope of Cluster 4 for CSF p-tau (Figure 4I; Supplementary Figure S2I) is unexpected. It may be possible that participants in Cluster 4 had the most severe cognitive impairment and the highest levels of CSF p-tau at baseline already, and thus the potential for CSF p-tau to further increase was limited, particularly at the later stages of the disease. Individuals in Cluster 4 who showed the most aggressive cognitive trajectory may also suffer from the most severely damaged blood–brain barrier (BBB), which could lead to the outflow of proteins from the CSF to the blood (Sweeney et al., 2018). However, the relationship between BBB integrity and AD pathologies is complex and not yet fully understood, and further research on this is needed. Further, the negative slope observed in this cluster could potentially be influenced by the limitations of a small sample size and short follow-up time. These factors may introduce variability errors, and thus, caution is warranted in interpreting the results. With respect to the markers presumed to represent neurodegenerative changes, our analyses found a more tightly coupled association between rates of cognitive decline and the magnitude of change in neurodegenerative biomarkers (i.e., aVV, aHV, and FDG SUVRs; see Figures 4D–F and Supplementary Figures S2D–F). Across these three neuroimaging markers, however, the magnitude of the associations of rates of cognitive decline with changes in these markers was distinct. For instance, all four cognitive trajectories exhibited significantly different slopes for ventricular enlargement, while the rates of hippocampal atrophy or brain hypometabolism of these four cognitive trajectories were not all different for each pairwise comparison, suggesting that ventricular enlargement is more closely coupled with cognitive changes than hippocampal atrophy or hypometabolism and thus maybe a more downstream event that follows hippocampal atrophy or hypometabolism (Jack et al., 2004, 2013). With regard to other cognitive and functional measures, as shown in Figures 4A–C and Supplementary Figures S2A–C (clusters 1 to 4 represented incremental rates of cognitive decline), rates of cognitive progression of the four clusters were the most closely coupled with changes in other cognitive and functional measures over time, as measured by MMSE, CDR-SB, and FAQ. These findings further supported the robustness of our clustering results as the pattern of the four cognitive trajectories was similar across distinct cognitive measures.

Several limitations of this study should be taken into account when interpreting our results. First, the individual trajectories on the ADAS-Cog-13 among patients with early AD were highly variable, as shown by the thin grey lines in Figure 2A. The cluster analysis used in this study should be interpreted as an exploratory analysis, rather than a confirmatory one. Indeed, it is possible that some patients could be categorized into a different cluster if the number of clusters changes. In this study, the 4-cluster solution was selected according to several considerations, including the BIC, the elbow method, and ensuring that each cluster had an adequate sample size. We acknowledge that a larger sample size of our study sample, especially the cluster 4 group, would be needed to yield more robust and generalizable findings. However, our linear mixed effects models with distinct rates of cognitive changes as the independent variable and other cognitive and functional outcomes (i.e., MMSE, CDR-SB, and FAQ) yielded a very consistent profile of cognitive decline across different cognitive assessments (Figures 4A–C and Supplementary Figures S2A–C), strengthening the notion that the four cognitive trajectories identified in the cluster analysis were stable and robust. Second, in the cluster analysis, the longitudinal measurements on ADAS-Cog-13, rather than other AD markers, were used as our clustering variable due to the fact that the primary objective was to investigate the variations in cognitive decline among patients with early AD, and the ADAS-Cog-13 is one of the most commonly used assessment to track cognitive progression in AD clinical trials. Third, although changes in AD biomarkers over extended periods (e.g., several decades) are nonlinear (Jack et al., 2013), our linear mixed-effects models assumed that they were linear. However, during shorter periods (e.g., within a period of 5-year follow-up), AD biomarker changes can be modeled as linear functions because the nonlinearity appears to be minimal (Luo et al., 2022). The fourth limitation of our study is that our analysis was based on the ADNI cohort, a highly educated sample, with limited diversity with respect to racial and ethnic characteristics. This has restricted the generalizability of our findings, and thus independent and large cohorts, population-based studies in particular, are needed to replicate and validate our results. However, the ADNI study was designed to represent a potential AD clinical trial population, and, in this study, we applied a largely similar inclusion and exclusion criteria compared to those used in recent and current clinical trials of early AD. Hence, our results may seem to be relevant in the context of early AD clinical trials. Fifth, it could be argued that our data-driven approach was capturing disease stages instead of different rates of cognitive trajectories based on the observation that these four clusters had different intercepts. It should be noted that our strategy to ensure a similar disease stage was to select individuals with similar baseline levels of cognitive performance, as reflected by the inclusion criteria of a global CDR score of 0.5 or 1 and an MMSE score of 20–30. This group of individuals included in the current study is classified as early AD and is also commonly used in clinical trials (van Dyck et al., 2023). Furthermore, the observation that baseline cognitive performance was an important predictor for future cognitive decline is consistent with previous findings (Schaeverbeke et al., 2021). Therefore, it would be not unexpected to observe that those with more severe cognitive impairment at baseline are also more likely to decline in the future. However, worse cognitive performance at baseline does not necessarily determine future cognitive decline, as there are several other variables (Table 3) or unobserved factors that also contribute to the variance in cognitive decline. For example, 22 out of 141 individuals in the Cluster 2 were diagnosed as mild AD at baseline, while 40 out of 108 individuals in the Cluster 3 were diagnosed as MCI at baseline (Figure 2D). Finally, our decision to combine MCI and mild AD dementia into a single group was primarily driven by the recent success of clinical trials in the field of AD, which achieved statistical significance (Sims et al., 2023; van Dyck et al., 2023). These trials employed similar inclusion criteria, notably combining individuals with MCI and mild AD dementia who are amyloid-positive into an “early AD” group. Motivated by these studies, we undertook the present study to delve deeper into the heterogeneity of cognitive decline within this participant group. Our goal was to potentially refine the recruitment process, thereby enhancing the likelihood of successful trials targeting patients in the early stages of AD.

Our study emphasizes the relevance of considering the heterogeneity of disease progression in early AD and provides further understanding of the temporal sequence of changes in AD biomarkers that correspond to different rates of cognitive decline. These findings have practical implications for the stratification of patients with early AD, potentially increasing the likelihood of identifying effective treatments for future AD clinical trials.

## Data availability statement

The datasets presented in this article are not readily available because data used in the present study has been made publicly available by the ADNI in the Laboratory of Neuro Imaging (LONI) database (https://adni.loni.usc.edu/). The authors of this work are not authorized to share the data. Requests to access the datasets should be directed to https://adni.loni.usc.edu/.

## Ethics statement

The studies involving humans were approved by each ADNI participant or authorized representative provided written informed consent and the institutional review board of each participating ADNI site approved the ADNI study. This study was conducted in accordance with the Declaration of Helsinki. This project was also submitted for review to the Institutional Review Board of Wenzhou Seventh People’s Hospital. However, since the study did not involve direct contact with human subjects and utilized de-identified data, the Institutional Review Board of Wenzhou Seventh People’s Hospital determined that formal review was not required. The studies were conducted in accordance with the local legislation and institutional requirements. The participants provided their written informed consent to participate in this study.

## Author contributions

XW: Investigation, Methodology, Validation, Visualization, Writing – original draft. TY: Formal analysis, Investigation, Methodology, Validation, Visualization, Writing – original draft. DJ: Investigation, Validation, Visualization, Writing – original draft. WZ: Conceptualization, Formal analysis, Investigation, Methodology, Project administration, Supervision, Validation, Visualization, Writing – review & editing. JZ: Conceptualization, Data curation, Formal analysis, Investigation, Methodology, Project administration, Supervision, Validation, Visualization, Writing – original draft, Writing – review & editing.

## Footnotes

^1^ https://adni.loni.usc.edu/

^2^ www.adni-info.org
