# Supplementary figures and images for: Characterizing the clinical heterogeneity of early symptomatic Alzheimer’s disease: a data-driven machine learning approach
Source: Front Aging Neurosci. 2024 Aug 12;16:1410544. doi: 10.3389/fnagi.2024.1410544 (PMC11348433; doi:10.3389/fnagi.2024.1410544)

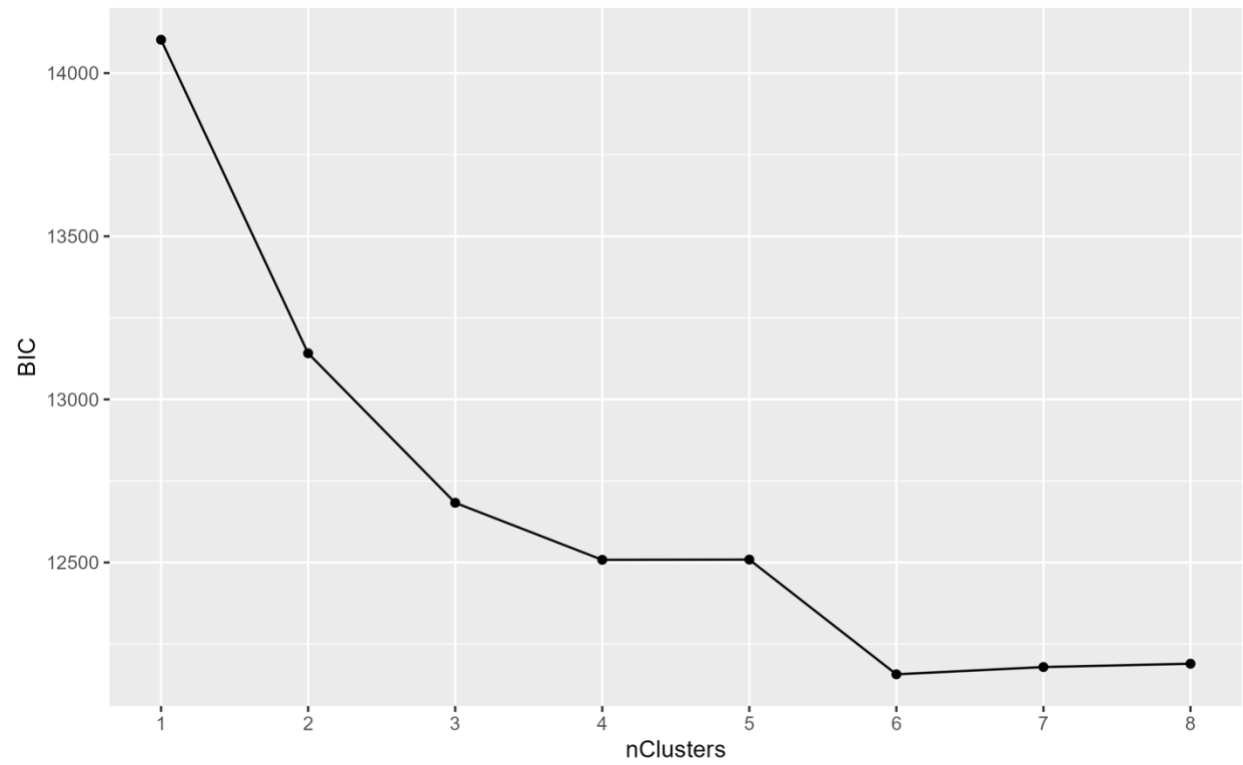

Fig S1. The elbow plot showing the BIC per cluster solution.

Supplement: Supplementary file 1 [file Data_Sheet_1.PDF]
