# Supplementary material for: Characterizing the clinical heterogeneity of early symptomatic Alzheimer’s disease: a data-driven machine learning approach
Source: Front Aging Neurosci. 2024 Aug 12;16:1410544. doi: 10.3389/fnagi.2024.1410544 (PMC11348433; doi:10.3389/fnagi.2024.1410544)
Supplement: Supplementary file 2 [file Data_Sheet_2.PDF]

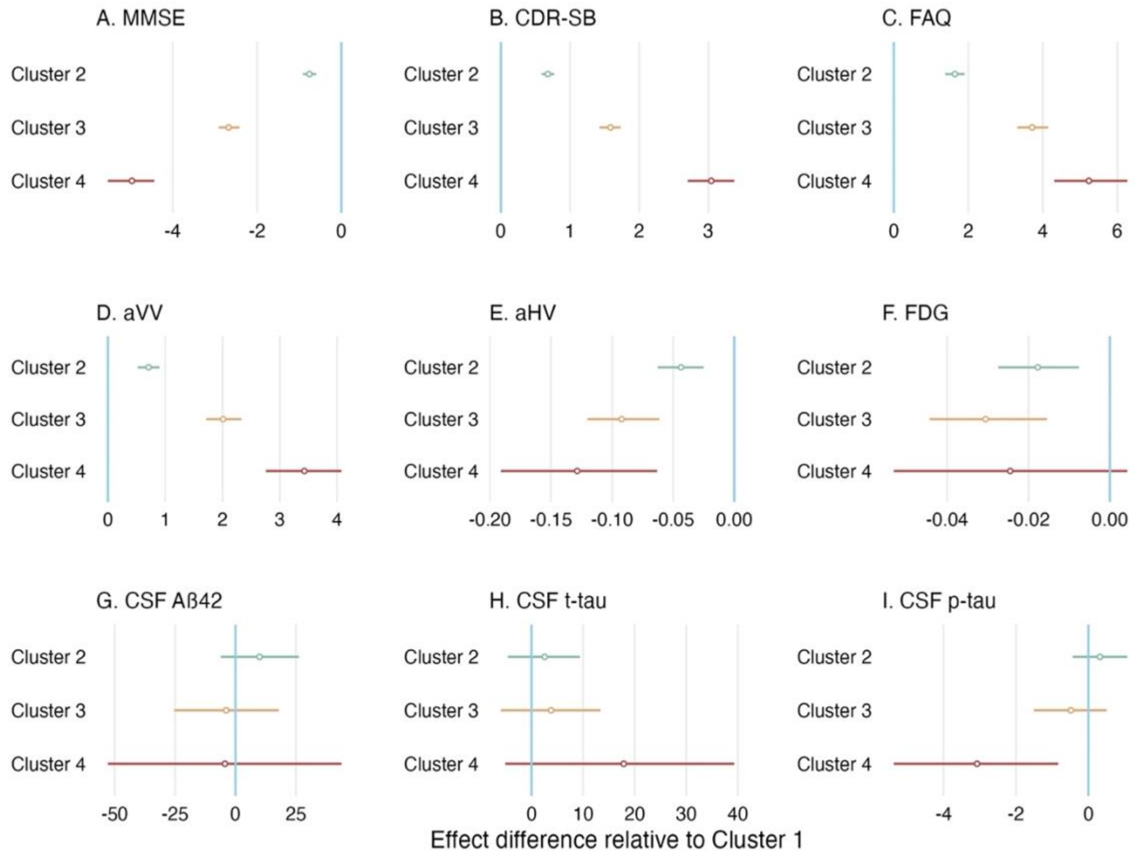

Fig S2. Forest plots showing effect difference relative to Cluster 1. Abbreviations: MMSE: Mini-Mental State Examination; CDR-SB: Clinical Dementia Rating-sum of boxes; FAQ: Functional Activities Questionnaire; aVV: Adjusted ventricular volume; aHV: Adjusted hippocampal volume; FDG: fludeoxyglucose; Aβ: β-amyloid, t-tau: total tau, p-tau: phosphorylated tau.
